# Nasal Acai Polysaccharides Potentiate Innate Immunity to Protect against Pulmonary *Francisella tularensis* and *Burkholderia pseudomallei* Infections

**DOI:** 10.1371/journal.ppat.1002587

**Published:** 2012-03-15

**Authors:** Jerod A. Skyberg, MaryClare F. Rollins, Jeff S. Holderness, Nicole L. Marlenee, Igor A. Schepetkin, Andrew Goodyear, Steven W. Dow, Mark A. Jutila, David W. Pascual

**Affiliations:** 1 Department of Immunology and Infectious Diseases, Montana State University, Bozeman, Montana, United States of America; 2 Rocky Mountain Regional Center for Excellence in Bioterrorism and Emerging Infectious Diseases, Colorado State University, Fort Collins, Colorado, United States of America; 3 Department of Microbiology, Immunology, and Pathology, Colorado State University, Fort Collins, Colorado, United States of America; National Institute of Allergy and Infectious Diseases, National Institutes of Health, United States of America

## Abstract

Pulmonary *Francisella tularensis* and *Burkholderia pseudomallei* infections are highly lethal in untreated patients, and current antibiotic regimens are not always effective. Activating the innate immune system provides an alternative means of treating infection and can also complement antibiotic therapies. Several natural agonists were screened for their ability to enhance host resistance to infection, and polysaccharides derived from the Acai berry (Acai PS) were found to have potent abilities as an immunotherapeutic to treat *F. tularensis* and *B. pseudomallei* infections. *In vitro*, Acai PS impaired replication of *Francisella* in primary human macrophages co-cultured with autologous NK cells via augmentation of NK cell IFN-γ. Furthermore, Acai PS administered nasally before or after infection protected mice against type A *F. tularensis* aerosol challenge with survival rates up to 80%, and protection was still observed, albeit reduced, when mice were treated two days post-infection. Nasal Acai PS administration augmented intracellular expression of IFN-γ by NK cells in the lungs of *F. tularensis*-infected mice, and neutralization of IFN-γ ablated the protective effect of Acai PS. Likewise, nasal Acai PS treatment conferred protection against pulmonary infection with *B. pseudomallei* strain 1026b. Acai PS dramatically reduced the replication of *B. pseudomallei* in the lung and blocked bacterial dissemination to the spleen and liver. Nasal administration of Acai PS enhanced IFN-γ responses by NK and γδ T cells in the lungs, while neutralization of IFN-γ totally abrogated the protective effect of Acai PS against pulmonary *B. pseudomallei* infection. Collectively, these results demonstrate Acai PS is a potent innate immune agonist that can resolve *F. tularensis* and *B. pseudomallei* infections, suggesting this innate immune agonist has broad-spectrum activity against virulent intracellular pathogens.

## Author Summary

Activation of the innate immune system offers an alternative and effective means to counter infection, particularly, in cases when the etiologic agent is unknown, such as a potential bioterrorism attack or when the agent is resistant to antibiotics. Here we report that a natural polysaccharide extract derived from the Acai berry (Acai PS) has potent abilities to counter infection when applied as a mucosal immunotherapeutic. Acai PS diminishes the replication of *F. tularensis* in human macrophages co-cultured with NK cells *in vitro*. In addition, nasal treatment of mice, prophylactically or therapeutically, with Acai PS results in significant protection against morbidity and mortality against pulmonary infection with virulent *F. tularensis* or *B. pseudomallei*. Of particular interest is that Acai PS utilizes the same mechanism of protection by enhancing Th1 cell immunity by both human and murine cells. Since an optimal Th1-type response is required for protection against a wide variety of infectious diseases, Acai PS represents a novel immunotherapeutic that could augment antibiotic therapy against a broad range of pathogens.

## Introduction

*Francisella tularensis* is a highly infectious, Gram-negative facultative intracellular bacterium that causes the zoonotic infection tularemia. *F. tularensis* infections can occur via insect bites, cutaneous contact with infected animal carcasses, ingestion of contaminated food and water, or inhalation of viable organisms [1]. The type and severity of tularemia depends on the strain, dose, and route of infection [2]. *F. tularensis* subspecies *tularensis* (type A) and *holarctica* (type B) cause the majority of human cases, with subspecies *tularensis* being more virulent [2]. Cutaneous tularemia is the most common form of human disease, but is rarely fatal [3]. Inhalation of *F. tularensis* results in respiratory or pneumonic tularemia and is most common in people in endemic areas who perform tasks that predispose them to infectious aerosols [2]. Untreated respiratory forms of disease have mortality rates of >30% [4], while antibiotic treatment can decrease this number to approximately 2% [5]. Pulmonary tularemia can present from a mild pneumonia to an acute infection with high fever, malaise, chills, cough, delirium, and pulse-temperature dissociation [2]. The high infectivity (10–50 microorganisms) [3] and mortality of *F. tularensis* infections have led to the weaponization of the organism, including the introduction of antibiotic resistance, by several nations [5]. Due to these concerns, *F. tularensis* has been determined to be a Category A Bioterrorism agent by CDC. No vaccines are currently licensed to prevent tularemia. Although a live vaccine strain (LVS) derived from *F. tularensis* subspecies *holarctica* was created over 50 years ago, questions remain regarding its efficacy and possible reversion to virulence, and it is not licensed for human use [2]. LVS is attenuated in humans, but remains virulent for mice, although it is not as virulent as wild-type A and B strains. As LVS causes a disease in mice that mimics tularemia in humans, it has been studied extensively as a model intracellular pathogen [6] and is utilized here as model to assay the efficacy of agonists to enhance resistance to *Francisella in vitro*, while our *in vivo* studies employ the fully virulent SchuS4 strain of type A *F. tularensis*.

*Burkholderia pseudomallei* and *B. mallei* are gram-negative facultative intracellular bacterial pathogens. *B. pseudomallei* is the etiologic agent of melioidosis and is endemic in parts of southeast Asia and northern Australia [7]. The clinical manifestations of melioidosis are protean and may vary from acute sepsis to chronic focal pathology and latent infection, which can reactivate decades later from an, as yet, unknown tissue reservoir [8]. Melioidosis can also mimic other infections such as glanders, typhoid fever, bacterial sepsis, and TB, depending on whether the disease is acute or chronic [8]–[10]. Community-acquired infection with melioidosis is most likely due to exposure to bacteria in soil or water through cuts or skin abrasions or via inhalation or ingestion [8]. No licensed prophylactic or therapeutic vaccine exists for *Burkholderia* infections, and *B. pseudomallei* is intrinsically resistant to a wide range of antimicrobial agents. In addition, prolonged antibiotic therapy (up to 6 months) is required to treat *Burkholderia* infections, and 10–15% of patients may relapse when antibiotic therapy is withdrawn [8], [11].

Due to the lack of efficacious vaccines and concerns about *F. tularensis* acquiring resistance to antibiotics via natural or illicit means and the intrinsic antimicrobial resistance of *B. pseudomallei*, we hypothesized that alternative immune or natural therapeutic-based intervention strategies could prove beneficial to augment current treatment regimens. Activation of the innate immune system can enhance resistance to a variety of bacterial and viral infections [6], 11–14. Immunotherapeutics may be particularly beneficial against diseases caused by intracellular pathogens since the antibiotics often recommended for treatment of these diseases, such as gentamicin, poorly penetrate host cells and therefore fail to reach the etiological agent of disease [14]. In situations where the etiological agent of disease is unknown, stimulation of innate immunity may also be useful since these immune responses are often capable of providing protection against a broad range of pathogens [6], [14]. To achieve this goal several natural agonists, including apple polyphenols (APP), amphotericin B (AmpB), securinine, Yamoa PS, and Acai PS were tested for their ability to enhance immunity to *F. tularensis* since each of these agonists has been previously shown to exhibit proinflammatory properties [12], [15]–[19].

Herein, we showed polysaccharides isolated from the Acai berry (Acai PS) enhanced clearance of *F. tularensis* from human macrophages upon co-culture with autologous natural killer (NK) cells. Mucosal administration of Acai PS also conferred both prophylactic and therapeutic protection against pulmonary *F. tularensis* and *B. pseudomallei* infections. The immunological basis for Acai PS-mediated protection both *in vitro* and *in vivo* is elucidated in this study.

## Results

### Acai PS and Yamoa PS enhance RAW264.7 macrophage clearance of *F. tularensis* LVS *in vitro*

**Figure 1:**
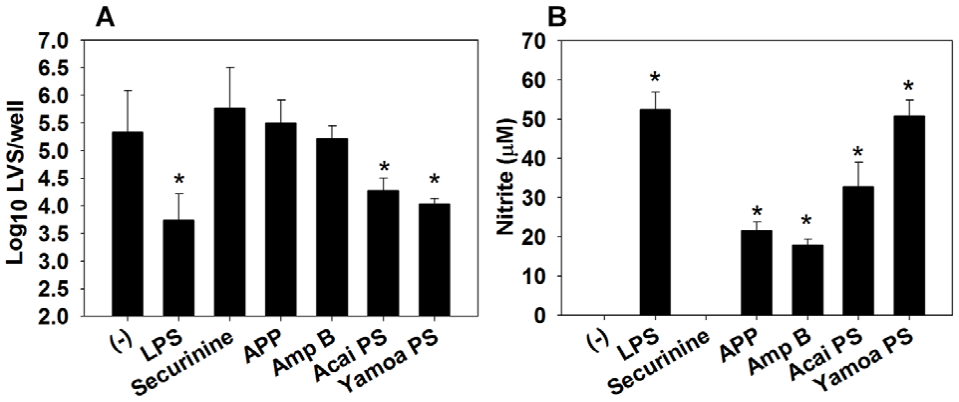
Natural agonists restrict replication of *F. tularensis* LVS in RAW264.7 macrophages. RAW264.7 macrophages (10^6^/well, 3 wells/treatment) were stimulated overnight (∼14 h) with 50 ng/ml LPS, 40 µM securinine, 40 µg/ml APP, 500 ng/ml amphotericin B, 10 µg/ml Acai PS, or 10 µg/ml Yamoa PS prior to infection with *F. tularensis* LVS (MOI∼300). After 20 h of infection, A) macrophages were lysed, and supernatants were diluted for CFU enumeration, and B) nitrite levels in the supernatants were determined. Error bars represent SD, * P<0.05, as compared to untreated cells. Data are representative of two independent experiments.

An initial screen of natural agonists for their ability to enhance macrophage resistance to *F. tularensis* infection was conducted. RAW264.7 cells, a murine macrophage-like cell line [20], were treated overnight prior to *F. tularensis* LVS infection. LPS (*E. coli* 0∶55, B5) was also included in our screen as a positive control for macrophage activation. Both intracellular bacterial burden and NO_2_ accumulation were measured (Figure 1). While amphotericin B (Amp B), Apple Polyphenol (APP; [21]), LPS, Acai PS, and Yamoa polysaccharides (Yamoa PS [15]) all enhanced nitric oxide (NO) production by RAW264.7 cells (Figure 1B), only LPS, Acai PS, and Yamoa PS significantly enhanced macrophage resistance to *F. tularensis* LVS (Figure 1A) at the indicated doses. Preliminary *in vivo* experiments indicated that only Acai PS was able to provide protection against pulmonary LVS challenge (data not shown). Yamoa PS previously was shown to induce strong reactivity to the Limulus Amebocyte Lysate (LAL) assay [15] and therefore was eliminated from further study. However, Acai PS has low amounts of endotoxin reactivity as measured by LAL assay, and its immunomodulatory effects are resistant to polymyxin B treatment [19]; therefore, it was selected for further evaluation.

### Acai PS upregulates macrophage surface activation molecule expression and stimulates proinflammatory cytokine production by mock- and LVS-infected RAW264.7 cells

To assess the immunomodulatory effects upon surface activation molecule expression by Acai PS treatment, RAW264.7 cells (originally derived from BALB/c mice) were treated with varying doses of Acai PS overnight prior to mock- or LVS- (Multiplicity of Infection [MOI]∼300) infection. RAW264.7 macrophages were then cultured for an additional 20 h prior to assessment of changes in surface activation molecule expression by flow cytometry, and cytokine and NO production were also measured in cell culture supernatants. Acai PS alone markedly stimulated CD40, CD80, and CD86 (Table S1). Subsequent LVS infection, Acai PS enhanced surface expression of CD11b, CD40, CD80, CD86, TLR2, and MHC class II in a dose-dependent fashion, while TLR4 expression was downregulated in both mock- and LVS- infected macrophages (Table S1). Acai PS also enhanced generation of NO, TNF-α, and IL-6 in a dose-dependent manner by RAW264.7 cells (Table S2) and also induced trace amounts of IL-1β and IL-12p40 (<300 pg/ml, data not shown).

### Acai PS mediates clearance of type A *F. tularensis* in RAW264.7 cells, but not in primary murine bone marrow-derived macrophages (BMDMs) via NO

To investigate the mechanism by which Acai PS enhances RAW264.7 cell resistance to *F. tularensis* infection, RAW264.7 cells and BMDMs derived from BALB/c mice were treated with varying amounts of Acai PS before infection with *F. tularensis* SchuS4. Pretreatment of RAW264.7 cells with as little as 10 µg/ml of Acai PS reduced SchuS4 replication, while the greatest protection was obtained using a 100 µg/ml dose (Table 1). Although the addition of 400 µM N^G^-Methyl-L-arginine (L-NMA), an inhibitor of NO production [22], did not totally abrogate NO production by RAW264.7 cells prestimulated with 100 µg/ml Acai PS (Table 2), L-NMA treatment did significantly diminish Acai PS-mediated resistance to *F. tularensis* SchuS4, while having no effect on unstimulated cells (Table 1); similar results were obtained using *F. tularensis* LVS (Figure S1). While Acai PS reduced intracellular replication of *F. tularensis* SchuS4 in RAW264.7 cells in an NO-dependent manner, Acai PS did not induce NO or enhance the clearance of *F. tularensis* SchuS4 from murine BMDMs (Table 1), which highlights the limitations of using cell lines as surrogates for primary cells. However, pretreatment of BMDMs with Acai PS did enhance phagocytosis of *F. tularensis* SchuS4 (Table 1). In addition, while infection of macrophages with strains of *Francisella* that do not cause disease in humans, such as *F. novicida*, results in rapid activation of the inflammasome and cell death [23], we did not find type A *F. tularensis* infection, or Acai PS to induce robust cytotoxicity of murine BMDMs or primary human macrophages at 20 h post-infection under the conditions tested (Table S3). This is in concordance with other studies that show type A *F. tularensis* does not vigorously activate the inflammasome in human dendritic cells [24].

**Table 1:** Acai PS enhances the clearance of type A *F. tularensis* from RAW264.7 cells, but not murine BMDMs via NO.

SchuS4-infected RAW264.7 cells (CFU burden)^a^				SchuS4-infected BMDMs (CFU burden)^a^		
Acai PS concentration				Acai PS concentration		
	Media	10 µg/ml	100 µg/ml	Media	10 µg/ml	100 µg/ml
0 hr	4.64 (0.14)	4.66 (0.11)	4.35 (0.14)	4.23 (0.26)	4.45 (0.06)	5.55 (0.11)*
4 hr	5.1 (0.05)	5.15 (0.09)	4.75 (0.06)*	4.92 (0.09)	5.09 (0.11)	5.82 (0.17)*
20 hr	6.12 (0.13)∧	5.55 (0.16)*	3.89 (0.18)* ∧	6.25 (0.14)	6.31 (0.02)	6.62 (0.20)
20 hr+L-NMA	6.01 (0.03)	ND	5.72 (0.05)*	6.13 (0.11)	ND	6.47 (0.19)

Cells were treated with Acai PS 16 hr prior to infection *F. tularensis* SchuS4 (MOI∼30), some wells were also pre-treated with 400 µM L-NMA.

a Log_10_ CFU/well from three wells/treatment shown; standard deviation in parentheses; results are representative of two independent experiments.

*: p<0.05 as compared to the same cell type not treated with Acai PS at the same time point.

**∧:** p<0.05 as compared to the same cell type, with the same Acai treatment, treated with L-NMA at 20 hr post-infection. ND = Not done.

**Table 2:** Acai PS induces TNF-α and NO in type A *F. tularensis*-infected RAW264.7 cells, but not in murine BMDMs.

SchuS4-infected RAW264.7 cells (NO) or (TNF-α)				SchuS4-infected BMDMs (NO) or (TNF-α)		
Acai PS concentration				Acai PS concentration		
	Media	10 µg/ml	100 µg/ml	Media	10 µg/ml	100 µg/ml
NO^a^	2.82 (0.19)	9.94 (0.38)*	58.6 (1.9)*	2.27 (0.19)	2.05 (0.16)	4.29 (0.38)*
NO+L-NMA^a^	1.87 (0.32)∧	ND	27.4(6.5)* ∧	1.52 (0.15)∧	ND	1.52 (0.31)* ∧
TNF-α^b^	2.1 (0.80)	4.1 (0.32)*	9.6 (1.5)*	0	0	0

Cells were treated and infected as in Table 1. 3/wells treatment at 20 hr post-infection shown; standard deviation in parentheses; results are representative of two independent experiments.

a Mean NO (µM) or

b TNF-α (ng/ml) production.

*: p<0.05 as compared to the same cell type not treated with Acai PS.

**∧:** p<0.05 as compared to the same cell type, with the same Acai treatment, treated with L-NMA at 20 hr post-infection. ND = not done.

### Acai PS treatment enhances the clearance of *F. tularensis* LVS from primary human macrophages co-cultured with autologous NK cells

**Figure 2:**
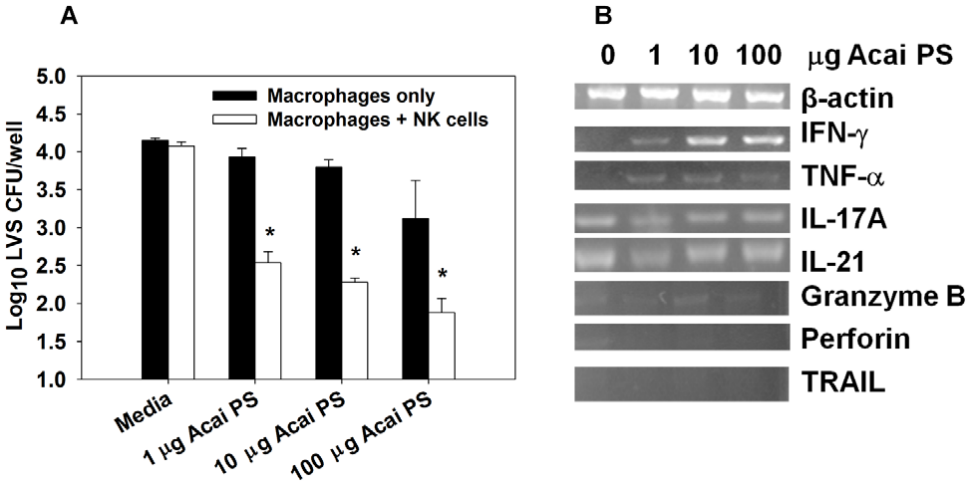
Acai PS enhances LVS clearance from in human primary macrophages and enhances NK cell IFN-γ mRNA. Human primary macrophages (1×10^4^cells/well, 3 wells/treatment) were derived from PBMCS and infected with LVS (MOI∼300). One day prior to macrophage infection, autologous NK cells were also isolated via magnetic sorting. Macrophages and NK cells were treated separately with varying amounts of Acai PS 16 h prior to macrophage infection. After infection of the macrophages, fresh media with or without Acai PS or fresh media containing NK cells (∼20 NK cells/macrophage) with or without Acai PS were then added to the macrophage containing wells. A) Twenty h after infection, NK cells (non-adherent) were removed, macrophages were lysed, and intracellular bacteria enumerated. Error bars represent standard error. *P<0.05, as compared to untreated macrophages. B) Total RNA was extracted from NK cells co-cultured with LVS-infected macrophages (with or without Acai PS), and RT-PCR was performed for β-actin (control) and IFN-γ, TNF-α, IL-17A, IL-21, granzyme B, perforin, and TRAIL. Results are representative of independent experiments from five different blood donors.

While Acai PS was unable to restrict the replication of *F. tularensis* in primary BMDMs, Acai PS previously was found to activate a variety of human leukocytes [19]. Therefore, we adopted a co-culture system in which primary human macrophages were infected with *F. tularensis* and co-cultured with autologous NK cells. Briefly, CD14^+^ cells were sorted and differentiated prior to Acai PS overnight treatment. Macrophages were infected with *F. tularensis* LVS and then cultured with or without purified autologous NK cells, some of which were also prestimulated with varying amounts of Acai PS overnight. CFU determinations were performed 20 h after infection, and total RNA was isolated from the NK cells at the same time. As little as 1 µg/ml of Acai PS was able to reduce LVS replication in macrophages co-cultured with autologous NK cells (Figure 2A). When Acai PS-treated macrophages were cultured without autologous NK cells, Acai PS-mediated protection occurred only at elevated concentrations (≥100 µg/ml) and varied from donor to donor (data not shown). While Acai PS was not found to augment IFN-γ mRNA expression by NK cells in the absence of infected macrophages (data not shown), Acai PS did enhance IFN-γ mRNA expression by NK cells co-cultured with *F. tularensis* LVS-infected macrophages (Figure 2B) in a manner inversely correlated with intracellular replication of LVS. Acai PS also augmented TNF-α mRNA by NK cells co-cultured with *F. tularensis* LVS-infected macrophages (Figure 2B); however, this effect was not consistent amongst all donors (data not shown). Acai PS was not found to consistently enhance mRNA's characteristic of cytotoxic activity (granzyme B, perforin, TRAIL) or the expression of IL-17 and IL-21 by NK cells co-cultured with *F. tularensis* LVS-infected macrophages (Figure 2B).

### Acai PS impairs *F. tularensis* SchuS4 replication in human primary macrophages co-cultured with NK cells via IFN-γ

**Figure 3:**
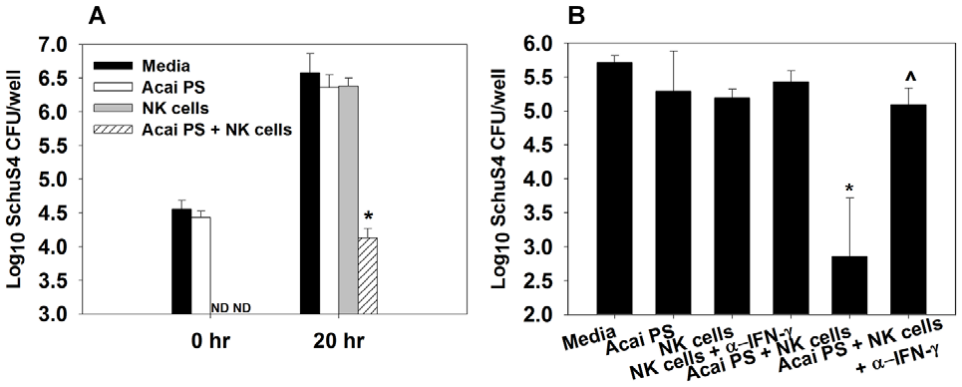
Acai PS reduces type A *F. tularensis* from primary human macrophages co-cultured with NK cells via IFN-γ. A) and B) Primary human macrophages (10^5^/well, 3 wells/treatment) and NK cells were isolated and treated separately with 100 µg/ml Acai PS 16 h prior to infection with *F. tularensis* SchuS4 (MOI∼30). Neutralizing anti-IFN-γ mAb, and/or autologous NK cells (∼5 NK cells/macrophage) were also added to some wells. Either immediately after (0 hr) or twenty h after infection, NK cells (non-adherent) were removed and A) macrophages were lysed and intracellular bacteria enumerated. Error bars represent standard deviation. *P<0.05 as compared to untreated macrophages. B) ∧P<0.05 as compared to Acai PS stimulated co-cultures neutralized of IFN-γ. Results are representative of independent experiments from three different blood donors. Similar results were obtained in macrophages infected with LVS (data not shown). ND = not determined.

Since Acai PS enhanced the resistance of human primary macrophages co-cultured with NK cells to *F. tularensis* infection, subsequent studies addressed the relevance of IFN-γ to this protection. Macrophages were prestimulated with Acai PS (100 µg/ml) overnight, infected with wild-type *F. tularensis* SchuS4 (MOI∼30), and then cultured with or without purified, autologous NK cells, some of which had been prestimulated with Acai PS (100 µg/ml) overnight. CFU determinations were performed at 20 h after macrophage infection. Similar to what was observed with LVS, Acai PS treatment of human macrophages alone had no effect on intracellular bacterial burden, while Acai PS treatment of macrophage/NK cell co-cultures reduced intracellular bacterial burden >100 fold without affecting phagocytosis (Figure 3A). Neutralization of IFN-γ completely ablated the protective capacity of Acai PS, while neutralization of IFN-γ in the absence of Acai PS or NK cells had no effect on intracellular bacterial replication (Figure 3B). The addition of 400 µM L-NMA to co-cultures treated with Acai PS had no effect upon bacterial replication, and NO was not detected via the Griess Reaction, indicating that the protective effect of IFN-γ induced by Acai PS is independent of NO production (data not shown).

### Acai PS enhances innate immunity to pulmonary type A *F. tularensis* infection

**Figure 4:**
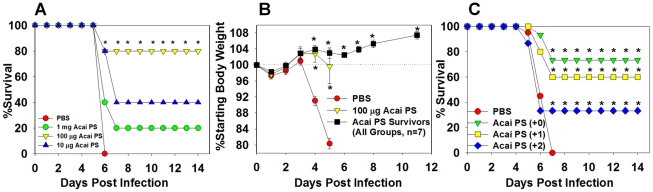
Nasal administration of Acai PS confers prophylactic and therapeutic protection against pulmonary Type A *F. tularensis* infection. Female C57BL/6 mice (5/group) were treated with PBS or with 10, 100, 1000 µg of Acai PS by the intranasal (i.n.) route one day prior to aerosol infection with *F. tularensis* SchuS4. A) Mice were monitored for morbidity and mortality twice daily for a period of 14–28 days, at which time survivors were euthanized, and B) body weights were monitored. C) Female C57BL/6 mice (n = 15–20/group) were i.n. treated with PBS or with 100 µg of Acai PS immediately after, one day after, or two days after aerosol infection with *F. tularensis* SchuS4. Mice were monitored for morbidity and mortality. *P<0.05 as compared to PBS group. Error bars depict S.D. Data depicted in C) are pooled from two independent experiments.

We previously found Acai PS to induce immunomodulatory effects when instilled into the lungs of naïve mice [19]. In particular, Acai PS was shown to induce IL-12, which is protective against *F. tularensis* LVS infection [25]. To assess whether Acai PS could confer protection against pulmonary infection with virulent *F. tularensis* SchuS4, C57BL/6 mice were treated nasally with 10, 100, or 1000 µg of Acai PS 24 h prior to aerosol infection with *F. tularensis* SchuS4, and changes in body weight and morbidity were recorded over time for up to 28 days after infection. Treatment of mice with 100 µg of Acai PS led to 80% survival, while 10 or 1000 µg Acai PS doses exhibited less potency (Figure 4A). Importantly, mice treated with Acai PS that survived infection showed negligible weight loss (Figure 4B) and clinical symptoms (data not shown); indicating Acai PS confers protection against both morbidity and mortality induced by virulent *F. tularensis* infection. Since the 100 µg dose of Acai PS was found to be optimal against aerosol challenge, in subsequent experiments mice were treated with 100 µg Acai PS at various time points after infection with *F. tularensis* SchuS4. When delivered by the intranasal (i.n.) route immediately after aerosol infection, Acai PS conferred 70–80% survival upon treated mice (Figure 4C), while all vehicle-treated animals succumbed to infection. Sixty percent of mice treated i.n. with Acai PS 24 h after aerosol challenge with *F. tularensis* SchuS4 survived, and even when Acai PS was given 48 h after infection, 33% of animals still survived (Figure 4C). As described above for prophylactic therapy, animals treated with Acai PS after aerosol infection that survived challenge displayed negligible weight loss and clinical symptoms (data not shown). Oral treatment of animals with Acai PS also conferred some level of protection against type A *F. tularensis* infection; however, this effect was variable (Table S4).

### Acai PS enhances a protective IFN-γ response during *F. tularensis* infection

**Figure 5:**
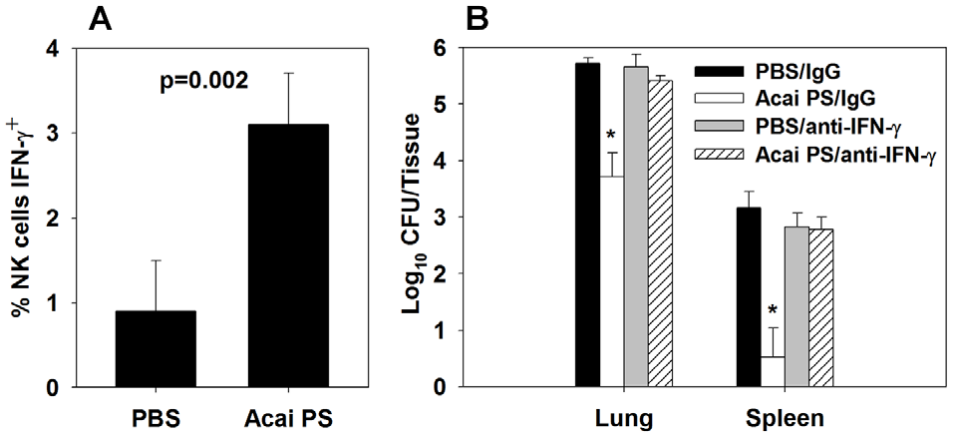
Acai PS enhances IFN-γ by innate leukocytes during pulmonary type A *F. tularensis* infection. C57BL/6 mice (n = 5/group) were treated i.n. with 1 mg of Acai PS one day prior to i.n. infection with 50 CFUs of *F. tularensis* SchuS4. Some mice were also depleted of IFN-γ two days prior to infection. A) Intracellular expression of IFN-γ was determined for lung NK cells by flow cytometry, and two days after infection, and B) lung and splenic bacterial burdens were determined. Data are representative of two independent experiments. Error bars depict SEM. *P<0.05 as compared to PBS-treated animals receiving the same antibody treatment.

To determine the mechanism by which Acai PS confers protection against *F. tularensis* infection, expression of intracellular IFN-γ by pulmonary leukocytes was assayed by flow cytometry. These studies utilized a 1000 µg Acai PS pretreatment, which we found to be optimal to protect against intranasal *F. tularensis* SchuS4 challenge (data not shown). The finding that a 1000 µg Acai PS dose was optimal against i.n. *F. tularensis* SchuS4 infection, while a 100 µg Acai PS dose was optimal against aerosol *F. tularensis* SchuS4 infection may reflect variations in the aerosol versus i.n. challenge models used in this study. We found i.n. pretreatment of mice enhanced intracellular expression of IFN-γ by NK T cells within two days after *F. tularensis* SchuS4-infection (Figure 5A). In addition, while Acai PS reduced bacterial burdens in the lungs and spleens of *F. tularensis* SchuS4, neutralization of IFN-γ abrogated this effect (Figure 5B–C).

### Acai PS enhances innate immunity to *B. pseudomallei* pulmonary infection and reduces bacterial replication and dissemination

**Figure 6:**
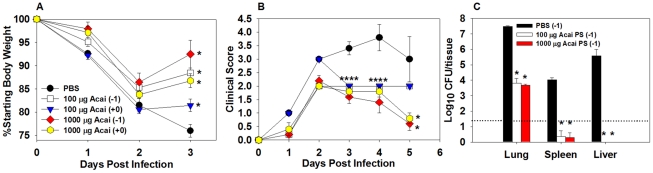
Nasal administration of Acai PS confers protection against pulmonary *B. pseudomallei* infection. Female C57BL/6 mice (n = 5/group) were treated i.n. with PBS or with 100–1000 µg of Acai PS one day prior to, or immediately after, intranasal infection with 3×10^3^ CFUs of *B. pseudomallei* 1026b. A) Body weights and B) clinical scores were recorded daily, and C) on day 3, CFU determinations were performed in the lungs, spleens, and livers. Error bars depict SEM. *P<0.05 as compared to PBS group. **** indicates that *P<0.05 for all Acai PS-treated groups in relation to PBS-treated group at this time point. Data depicted in A–B) are representative of two independent experiments. The dashed line in C) indicates the limit of bacterial CFU detection.

Stimulation of innate immunity with an immunotherapeutic such as Acai PS would be particularly valuable in situations where the etiological agent of disease is unknown, such as a bioterrorist attack, as induced innate immune responses are often capable of providing protection against a broad range of organisms. In addition, immunotherapy could be of particular benefit to counter infections due to bacteria that are intrinsically resistant to antibiotics, such as *B. pseudomallei*, a CDC Category B Bioterrorism agent. As Acai PS augmented immunity to *F. tularensis* infection, along with enhancing the expression of IFN-γ, which is crucial for protection from *B. pseudomallei* infection [26], we tested the effects of Acai PS against *B. pseudomallei* infection to assay whether Acai PS has potential as a broad spectrum therapeutic to combat pulmonary infections. C57BL/6 mice were treated i.n. with Acai PS prior to, or immediately after, i.n. infection with 3×10^3^ CFUs of *B. pseudomallei* 1026b. Body weights and clinical scores were recorded. I.n. treatment of mice with 100 or 1000 µg Acai PS 24 h prior to, or immediately after, *B. pseudomallei* infection resulted in significantly diminished weight loss and clinical scores (Figure 6A–B). Treatment of mice with ≤10 µg of Acai PS or treatment of mice with Acai PS (10–1000 µg) ≥24 h after *B. pseudomallei* infection did not result in significant protection (data not shown). Next, to determine the effects of Acai PS on bacterial colonization and dissemination, mice were treated i.n. with 100 or 1000 µg of Acai PS 24 h prior to i.n. infection with 3×10^3^ CFUs of *B. pseudomallei* 1026b. Bacterial burdens were determined in the lungs, spleens, and livers 72 h after infection. Treatment of mice with either dose of Acai PS reduced *B. pseudomallei* replication in the lungs by ∼10,000-fold (Figure 6C). Treatment of mice with Acai PS also reduced dissemination into peripheral tissues. *B. pseudomallei* CFUs were below the limit of detection (∼33 CFUs) in the spleens of 80% of animals treated with either dose of Acai PS, while no bacteria were recovered from the livers of any animals treated with either dose of Acai PS (Figure 6C). In addition, all mice treated prophylactically with 100 or 1000 µg Acai PS (n = 20) survived nasal infection with 3×10^3^ CFUs of *B. pseudomallei* 1026b; however, the lethality of this dose in control animals varied from 60–100% in different experiments (8/10 control animals succumbed to infection). While 100 and 1000 µg Acai PS doses conferred similar protection against challenge with 3×10^3^ CFUs of *B. pseudomallei*, a 1000 µg Acai PS provided the best protection against high dose i.n. challenge (1×10^4^ CFUs) with *B. pseudomallei* (Figure S2A–B). These results indicate that an elevated dose of Acai PS may be required against a high dose bacterial challenge in order to protect the host against a more acute disease.

### Acai PS enhances IFN-γ responses by NK cells and γδ T cells during pulmonary *Burkholderia* infection

**Figure 7:**
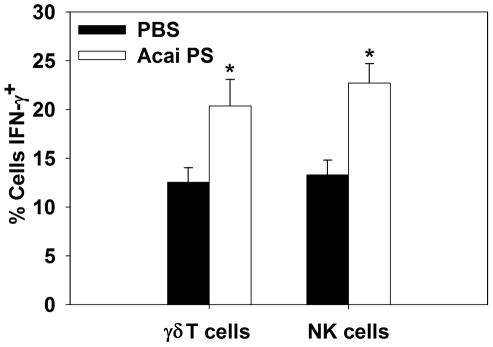
Acai PS augments IFN-γ responses by γδ T cells and NK cells during pulmonary infection. C57BL/6 mice (n = 5/group) were treated by the i.n. route with 1 mg of Acai PS one day prior to i.n. infection with 5×10^5^ CFUs of *B. thailandensis* E264. One day after infection, cells were harvested from lungs, and intracellular IFN-γ expression by γδ T cells and NK cells was determined by flow cytometry. Error bars depict SEM. *P<0.05 as compared to the same cell type from PBS-treated animals.

To assess the mechanism of protection mediated by Acai PS on innate lymphocytes during pulmonary infection, the *B. thailandensis* (BSL-1 strain) model of *Burkholderia* infection [27] was used. C57BL/6 mice were given Acai PS i.n. 24 h prior to i.n. infection with 5×10^5^ CFUs of *B. thailandensis* E264. Pulmonary NK and γδ T cells were then assayed 24 h after infection by flow cytometry for the intracellular expression of IFN-γ. Acai PS enhanced IFN-γ expression by both NK and γδ T cells in *B. thailandensis*-infected mice (Figure 7); indicating Acai PS can augment the IFN-γ responses of innate lymphocytes during pulmonary *Burkholderia* infection.

### Acai PS requires IFN-γ and NK cells to confer protection against pulmonary *B. pseudomallei* infection

**Figure 8:**
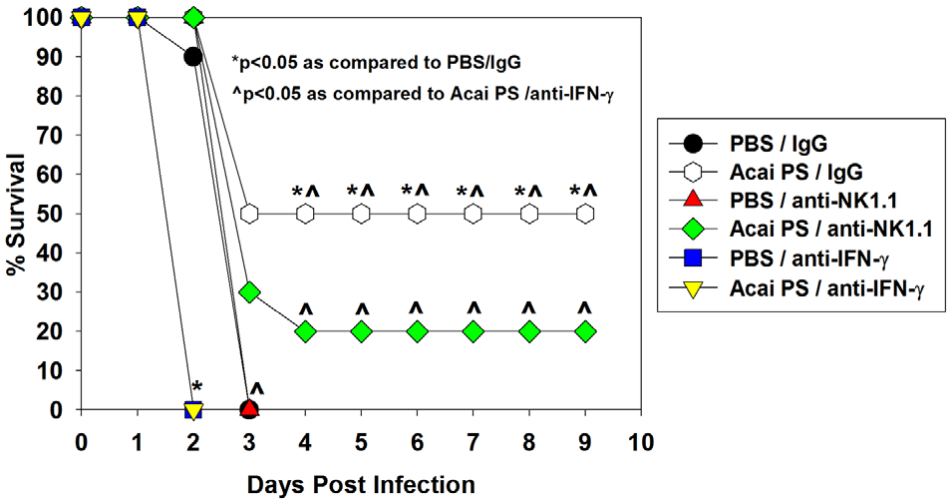
Acai PS requires IFN-γ and NK cells for optimum protection against *B. pseudomallei* infection. Two days prior to infection, C57BL/6 mice received rat IgG, anti-IFN-γ, or anti-NK1.1 mAb. Mice were treated i.n. with PBS (n = 5–10/group) or 1 mg of Acai PS (n = 10/group) one day prior to i.n. infection with 1×10^4^ CFUs of *B. pseudomallei* 1026b. Survival was monitored over time. *P<0.05 as compared to animals receiving PBS and IgG. ∧P<0.05 as compared to animals receiving Acai PS and anti-IFN-γ.

As Acai PS was found to enhance the pulmonary Th1-type response, which is critical for control of *Burkholderia* infections [11], 26,28, the role of Th1-type responses in Acai PS-mediated protection against *B. pseudomallei* infections was further investigated. For these studies, mice were treated i.n. with 1000 µg of Acai PS 24 h prior to infection. Some mice were also depleted of IFN-γ or NK cells via neutralizing antibody 24 h prior to Acai PS treatment (control animals received rat IgG). While the survival conferred by Acai PS in control animals was suboptimal against a high-dose challenge, Acai PS-mediated survival was totally ablated in IFN-γ-depleted mice and partially reduced in mice depleted of NK cells (Figure 8). In addition, while Acai PS mitigated clinical symptoms in *B. pseudomallei*-infected mice, this effect was abrogated in the absence of IFN-γ (data not shown). These results indicate that, similar to what was observed *in vitro* and *in vivo* with *F. tularensis*; Acai PS requires IFN-γ and possibly NK cells for protection against pulmonary infection with *B. pseudomallei*.

## Discussion

Enhancing innate immunity by agonist therapy could potentially augment resistance to infection and could also complement traditional vaccination and antibiotic strategies for treating infectious diseases [6], [11], [12], [14], [15]. In this study, the abilities of several natural agonists with immunomodulatory capabilities: APP, AmpB, securinine, Yamoa PS, Acai PS, and LPS [12], [15]–[18] were assayed for their ability to potentiate macrophage resistance to *F. tularensis* LVS infection. While APP, AmpB, Yamoa PS, and Acai PS each enhanced NO production by LVS-infected RAW264.7 macrophages, only Yamoa PS and Acai PS conferred significant resistance to LVS replication at the doses tested. LPS also enhanced NO production and LVS clearance, which was not surprising, as TLR4 agonists have been shown to increase resistance to infection with *F. novicida*, a strain of *Francisella* that is virulent for mice, but rarely causes disease in humans [29]. Yamoa PS was not further examined due to concerns about possible endotoxin contamination, presumably due to endophytic bacteria residing in bark [15], the source of this polysaccharide. In contrast, Acai PS contains low amounts of endotoxin (<0.01 EU/µg), has MyD88-independent effects, and has immunomodulatory effects resistant to polymyxin B treatment [19]. In addition, Acai PS is non-toxic to lymphocytes at concentrations up to 500 µg/ml [19] and is not found to have direct antibacterial (cytotoxic) effects against *Francisella* in PBS or cell culture media (data not shown).

Acai PS is derived from the berry of the palm tree *Euterpe oleracea* Mart. indigenous to the Amazon River basin in South America. This fruit is commonly used to make beverages and food additives and is used as a herbal medicine [30]–[34]. Biochemical studies reveal Acai contains numerous compounds, particularly anthocyanins, proanthocyanidins, and other flavonoids [34]. While many studies have focused on the antioxidant properties of Acai [33], [35]–[38] presumably attributable to its polyphenols and related classes of compounds, here we concentrated on the activities of Acai PS as the polysaccharide fraction, rather than the polyphenol fraction of Acai, induces a proinflammatory response [19]. We previously demonstrated that Acai PS stimulates both γδ T cells and myeloid cells *in vitro* and incites the recruitment of neutrophils and activates DCs/macrophages to the lung *in vivo* [19]. Therefore, since Acai PS has potent immunomodulatory activities and is effective at restricting the replication of *F. tularensis* LVS in RAW264.7 cells, it was investigated for its potential as an innate immune agonist.

In addition to augmenting the clearance of *F. tularensis* LVS and SchuS4 in RAW264.7 cells via NO, Acai PS also enhanced cell surface expression of CD11b, CD40, CD80, CD86, MHC class II, and TLR2 in a dose-dependent manner in both mock- and *F. tularensis* LVS infected-macrophages; however, TLR4 expression was downregulated. TLR4 expression has been shown to be downregulated following LPS stimulation [39], and while Acai PS is low in endotoxin (<0.01 EU/µg), is resistant to polymyxin B neutralization, and has MyD88-independent effects [19], it is possible Acai PS may still signal through TLR4 via an alternative mechanism such as TRIF [40].

While Acai PS was able to reduce the intracellular replication of *F. tularensis* in RAW264.7 cells, Acai PS was not found to induce NO or restrict the replication of *F. tularensis* in primary human macrophages or murine BMDMs. This finding is presumably due to the fact that primary cells and, in particular human macrophages, do not produce NO as readily as do macrophage cell lines [41], and such findings stress that cell lines are not always a suitable surrogate for primary cells. While Acai PS did not enhance the clearance of *F. tularensis* in macrophages alone, Acai PS can also activate innate lymphocytes in addition to macrophages [19]. Therefore, we adapted a co-culture system in which we tested the effect of Acai PS treatment on human monocyte-derived macrophages (cultured with or without autologous NK cells) infected with *F. tularensis*. While Acai PS was not able to directly stimulate human primary macrophages for clearance, a ∼100–1000-fold reduction in replication occurred when macrophages were co-cultured with autologous NK cells. Others have shown murine NK cells stimulated *in vivo* could impair intracellular replication of *F. tularensis* LVS *in vitro* [42], and depletion of NK cells reduces the time to lethality during pulmonary infection [43]. Of particular interest is that NK cells are a major source of IFN-γ in pulmonary tularemia [44]. RT-PCR analysis revealed Acai PS stimulated human NK cells co-cultured with LVS-infected macrophages possessed elevated levels of IFN-γ mRNA, while neutralization of IFN-γ *in vitro* diminished the protective effect of Acai PS in macrophages infected with *F. tularensis* LVS or SchuS4. NO was not detected in cell culture supernatants from our human macrophage/NK cell co-cultures, and iNOS inhibition had no effect on replication, indicating the protection conferred by Acai PS-induced IFN-γ in the human co-culture model is NO-independent, similar to what others have described for IFN-γ treated macrophages infected with *F. tularensis* SchuS4 [45]. Treatment of NK cells in the absence of *Francisella*-infected macrophages did not result in robust induction of IFN-γ mRNA, indicating there may be a synergetic effect of Acai PS and infection upon NK cells. Our previous finding that Acai PS induces IL-12 *in vivo* may indicate the macrophage is responsible for IL-12 production, which in turn induces IFN-γ mRNA by the NK cell. Indeed, neutralization of IL-12 *in vitro* did reduce IFN-γ mRNA by NK cells in some co-cultures treated with Acai PS (data not shown). While it is known human NK cells can enhance the clearance of intracellular organisms, such as *Brucella* in autologous macrophages via contact-dependent, cytotoxic mechanisms [46], the effect of Acai PS on NK cell mediated-cytoxicity may be minimal, since marked differences in the mRNA expression for perforin, granzyme B, or TRAIL were not observed by Acai PS-treated NK cells co-cultured with autologous LVS-infected macrophages.

As IFN-γ was induced by Acai PS *in vitro*, and is essential for protection against experimental tularemia [43], we assessed whether Acai PS could confer protection against *in vivo* challenge by employing an aerosol model of type A *F. tularensis* infection thought to most mimic human disease [47]. We utilized *F. tularensis* SchuS4 for all our *in vivo* infections, because, while *F. tularensis* LVS is widely used as a model organism to study immunological responses [48], emerging evidence suggests the *in vivo* immune response differs between SchuS4 and LVS [24], [47], [49], and immunotherapeutic strategies that confer potent protection against pulmonary LVS infection only confer partial or negligible protection against pulmonary infection with SchuS4 [6], [50], [51]. Since Acai PS enhanced the clearance of *Francisella* in murine macrophages and in human macrophages co-cultured with NK cells, and because Acai PS had potent immunomodulatory effects in the lung [19], Acai PS was tested as a mucosal immunotherapeutic to treat pulmonary type A *F. tularensis* infections. It was found that i.n. pretreatment of mice with Acai PS conferred up to 80% protection against *F. tularensis*-induced mortality, which, to our knowledge, is the highest degree of protection demonstrated by an immunotherapeutic and also represents the first mucosal immunotherapeutic to confer significant survival against pulmonary type A *F. tularensis* infection. Importantly, Acai PS provided significant protection when administered i.n. within 48 h after pulmonary infection and thus is the first immunotherapeutic demonstrating post-exposure protection of any kind against pulmonary type A *F. tularensis* infection. Acai PS was also able to reduce bacterial burdens in the lungs and spleens of mice infected with *F. tularensis* SchuS4. In addition, similar to what was observed *in vitro* in human cells, Acai PS augmented IFN-γ expression by NK cells in the lungs of treated animals infected with *F. tularensis* SchuS4, while neutralization of IFN-γ abrogated the protective effect of Acai PS. The finding that Acai PS is able to protect against infection even when administered one or two days after infection, at which time SchuS4 is already present in the spleen and liver, is intriguing. Mucosal administration of therapeutics can have systemic effects, and compounds delivered nasally enter the bloodstream. In preliminary studies, we have found that mucosal administration of Acai PS enhances serum levels of TNF-α (Holderness et al, manuscript in preparation). As TNF-α is protective against tularemia, it is possible that nasal administration of Acai PS also has an effect against systemic replication of *Francisella*, which may account for the post-exposure protection conferred by Acai PS observed here. Acai PS is also heat-resistant, and we have found it to have potent protective effects after shipment at ambient temperature, as demonstrated by the protection observed in Figure 6A–C. Therefore, since Acai PS does not require refrigeration (cold-chain management) and adapts a needle-free mucosal method of administration, it offers a practical strategy during emergencies, such as pandemics or bioterrorist attacks, when expeditious treatments of the affected populace would be required [52].

The downregulation of TLR4 by Acai PS observed by flow cytometry indicated Acai PS may signal at least partially through TLR4. However, work by others indicates TLR4 stimulation alone is an insufficient method to protect against experimental tularemia, particularly, when administered after infection. Nasal administration of a TLR4 agonist prior to, but not after, pulmonary infection with *F. novicida* could confer protection [29], while intraperitoneal administration of a TLR4 agonist could confer some level of protection when given 48 h before pulmonary infection with type A *F. tularensis* [51]; however, this effect is diminished when the TLR4 agonist was given only at the time of infection. In addition, others have demonstrated that pulmonary administration of LPS has minimal effects upon the immune response when given 24 h after type A *F. tularensis* infection [53], indicating that type A *F. tularensis* infection actively suppresses TLR4 signaling. Since we found Acai PS has potent protective effects when given ≥24 h after infection, it would appear that Acai PS also signals through a receptor in addition to TLR4, and the low levels of LPS present in Acai PS are not responsible for the observed protection. Botanical polysaccharides are known to signal through a variety of receptors, including TLRs and carbohydrate receptors [54]. Work on the receptors utilized by Acai PS is ongoing in a separate study, but Acai PS appears to require both TLR4/TRIF along with carbohydrate receptors (Holderness *et al*, manuscript in preparation) to mediate its effects. Therefore, future studies of the receptors required for Acai PS-mediated signaling and protection could reveal receptors to be targeted for immunotherapy against *F. tularensis* and other diseases.

Stimulation of innate immunity with an immunotherapeutic such as Acai PS would be particularly valuable in situations where the etiological agent of disease is unknown, as induced innate immune responses are often capable of providing protection against a broad range of organisms [6]. In addition, immunotherapy could be of particular benefit to counter bacterial infections intrinsically resistant to antibiotics. To this end, we tested Acai PS against pulmonary infection with *B. pseudomallei*, an organism intrinsically resistant to antibiotics, to determine if Acai PS has potential as a broad spectrum immunotherapeutic. We found Acai PS enhanced immunity to *B. pseudomallei* when given prior to, or immediately after, infection. Acai PS also potently restricted *B. pseudomallei* replication within the lungs and dissemination to peripheral tissues.

To assess the effects of Acai PS on innate leukocytes during infection, we assayed the expression of IFN-γ in leukocytes from the lungs of mice infected with *B. thailandensis*. We found Acai PS augmented the expression of IFN-γ in both NK and γδ T cells from *B. thailandensis*-infected animals, indicating an enhanced Th1-type response of these cell types. Since Acai PS also enhanced the IFN-γ response of human and murine NK cells during *in vitro* and *in vivo* models of *F. tularensis* infection, the role of NK cells and IFN-γ in Acai PS-mediated protection against *B. pseudomallei* was assessed. As a result, IFN-γ was entirely responsible for Acai PS-mediated protection, while NK cells were also required to some extent. These results demonstrate Acai PS mediates protection against infection in human cells *in vitro* and in *in vivo* murine models in a similar manner as NK cells and IFN-γ are required for protection in both systems, indicating our protective effects *in vivo* with mice have relevance to humans. As neutralization of NK cells did not entirely ablate protection against *B. pseudomallei in vivo*, it is possible the effects of Acai PS on other cells are also required for protection. We have previously found Acai PS to stimulate human γδ T cells *in vitro* [19], and here we show Acai PS augments the Th1-type responses of γδ T cells in infected lungs; therefore, as γδ T cells are known to confer protection against a number of intracellular pathogens such as *Brucella* and *Listeria* [55], [56], future studies will investigate the role of γδ T cells in Acai PS-mediated protection. In a clinical setting, an immunotherapeutic such as Acai PS would most often be used in conjunction with antibiotics. Recent studies have demonstrated immunotherapy can synergize with antibiotic therapy of bacterial infections, including *Burkholderia* [26]; therefore, additional studies will assess the effects of Acai PS in combination with antibiotic therapy.

In summary, we show immunotherapy with natural agonists such as Acai PS is an effective means to confer protection against bacterial infection. In fact, Acai PS appears to be the most potent immunotherapeutic reported to date to combat pulmonary type A *F. tularensis* infections and is also the first one demonstrated to confer significant survival when given mucosally or after infection. Of particular interest is Acai PS was also able to confer protection against pulmonary infection with both *F. tularensis* and *B. pseudomallei*, as previous studies demonstrated immunotherapeutics that induce potent protection against *B. pseudomallei* may only confer partial or negligible protection against type A. *F. tularensis* [6], [11], [50], indicating Acai PS has broad spectrum immunotherapeutic potential to combat intracellular bacterial infections. Acai PS also enhanced the Th1 cell response of innate leukocytes during infection both *in vivo* and in human cells. As optimal Th1 cell immunity is required for protection against a broad range of infections, Acai PS should be investigated as a possible immunotherapy that could augment or complement traditional antibiotic and vaccination strategies against a range of pathogens.

## Materials and Methods

### Ethics statement

All animal care and procedures were in accordance with the recommendations in the Guide for the Care Use of Laboratory Animals of the National Institutes of Health. All animal protocols were approved by Institutional Animal Care and Use Committees at Montana State University (protocol approval: 2009-27, 2011-25) or Colorado State University (protocol approval 09-001) and all efforts were made to minimize suffering. Human subjects testing was performed in accord with the Institutional Review Board of Montana State University (protocol approval: JS072809), and written, informed consent was obtained from all individuals.

### Bacterial strains, culture conditions and mice

*F. tularensis* SchuS4 or LVS was cultured in modified Mueller–Hinton (MMH) broth (0.025% ferric pyrophosphate, 2% IsoVitaleX and 0.1% glucose) at 37°C with constant shaking overnight, aliquotted into 1 ml samples, frozen at −80°C, and thawed just before use, as previously described [57]. Frozen stocks were titrated by enumerating viable bacteria from serial dilutions plated on MMH agar (0.025% ferric pyrophosphate, 2% IsoVitaleX, 0.1% glucose, and 0.025% fetal bovine serum). The numbers of viable bacteria in frozen stock vials varied by less than 5% over a 10 month period. These stocks were used to generate cultures for *F. tularensis* SchuS4 or LVS infection studies. Frozen stocks of *B. pseudomallei* of known titers were prepared from cultures grown in Luria-Bertani (LB) broth (BD Biosciences, San Jose, CA) by freezing the cultures in LB medium containing 20% glycerol. Inocula for *in vivo* infection with *B. pseudomallei* were prepared by thawing and diluting frozen stocks in sterile PBS [11]. All experiments with *F. tularensis* SchuS4 or *B. pseudomallei* 1026b were performed in biosafety level 3 facilities at Montana State University or Colorado State University. *Burkholderia thailandensis* E264 was acquired from ATCC (Manassas, VA). Prior to infection, *B. thailandensis* were grown from frozen glycerol stock in LB at 37°C overnight and freshly diluted 1∶100 into 100 ml of LB. The bacteria were grown to an optical density (OD_600_) of 1.9 (∼1×10^9^ cfu/ml) and diluted in PBS prior to infection [27].

Six-week-old female C57BL/6 or BALB/c mice were purchased from Charles River Laboratories. All mice were housed in sterile microisolater cages in the laboratory animal resources facility at Montana State University or the Biohazard Research Building BSL-3 facility at Colorado State University and were provided with sterile water and food ad libitum.

### Acai PS preparation

Acai fruit pulp was obtained from Acai Berry Pure (Acai Berry Pure Bulk; Carlsbad, CA). Polysaccharides were isolated from this powdered Acai, as described previously [15], [19]. Briefly, 1500 g of Acai powder was extracted with 8 liters boiling distilled H_2_O for 1 h. The aqueous extract was then centrifuged at 2,000× g for 15 min, and a 4-fold volume of ethanol was added to the supernatant to precipitate polysaccharides overnight at 4°C. The precipitate was pelleted by centrifugation, re-dissolved in distilled H_2_O and centrifuged at 2,000× g for 15 min. The supernatant fluid (crude polysaccharide extract) was fractionated using ion-exchange chromatography on a DEAE-cellulose column equilibrated with 0.05M Tris-HCl buffer (pH 8.0). Bound material was sequentially eluted with 0.05M Tris-HCl buffer and 2M NaCl. The presence of polysaccharides in the unbound fraction, eluted with 0.05M Tris-HCl buffer, was minimal (<0.1% of total bound fraction). The Acai-PS fraction was generated from this preparation after concentration in an Amicon concentrator with a 10 kDa Amicon PM10 membrane (Millipore; Billerica, MA). This preparation yields a fraction that is >99% carbohydrate and >92% polysaccharides. Monosaccharide analysis reveals that Acai PS consists primarily of arabinose, galacturonic acid, and galactose [19]. Endotoxin levels were determined using the LAL assay, as described [19]. Endotoxin levels for the Acai PS used in this study were <0.01 EU/µg.

### Generation of bone marrow-derived macrophages

Bone marrow-derived macrophages (BMDM) were generated by flushing the bone marrow from the femurs of BALB/c mice with RPMI 1640 media. Freshly collected bone marrow cells were cultured overnight in complete media (CM; RPMI 1640, 10% fetal bovine serum [Atlanta Biologicals, GA], 10 mM HEPES buffer, 10 mM nonessential amino acids, 10 mM sodium pyruvate) containing 5 ng/ml recombinant murine M-CSF (Peprotech, Rocky Hill, NJ). The non-adherent cells were then collected and cultured for an additional six days in CM with 30 ng/ml M-CSF to generate macrophages.

### Infection of RAW264.7 cells and murine BMDMs

Murine BMDMs or RAW264.7 macrophages were seeded at 1×10^6^ cells/well in CM without antibiotics in 24-well microtiter plates (BD Labware, Franklin Lakes, N.J.) at 37°C/5% CO_2_ prior to infection. Macrophages were infected with *Francisella tularensis* LVS at an MOI of ∼300 or *F. tularensis* SchuS4 at an MOI of ∼30 for two h at 37°C. Cells were then washed once with PBS, and then fresh CM containing 50 µg/ml gentamicin were added to each well, and cells were incubated for 30 min at 37°C to kill extracellular bacteria. Cells were then washed twice with PBS, and then fresh complete media without antibiotics were added to the wells for the remainder of the experiment (this is considered the “0 hour” time point). For time points of >8 h, gentamicin was added to the wells for the last 45 min of incubation. To enumerate intracellular bacteria, cells were washed three times with PBS and then lysed with sterile deionized water. Serial logarithmic dilutions of macrophage lysates were then performed and plated in triplicate onto MMH agar for incubation at 37°C/5% CO_2_ for 2–3 days. In some cases, macrophages were stimulated at various time points before or after infection with varying concentrations of agonist. In addition, L-NMA (Sigma-Aldrich, St. Louis, MO) was added to selected wells to inhibit NO production. Supernatants were collected and frozen until analysis by cytokine ELISA or the Griess reaction.

### Cytotoxicity, cytokine, and NO_2_^−^ production assays

Supernatants from *Francisella*-infected RAW264.7 and human macrophages were collected at various time points and measured for cell death, production of cytokines and, the oxidized product of NO. Cell death was determined by measuring lactate dehydrogenase LDH release using a cytotoxicity detection kit according to manufacturer's instructions (Roche, Indianapolis, IN). Cytokine-specific ELISAs were performed, as described previously [58], [59]. All NO_2_^−^ detection chemicals were obtained from Sigma-Aldrich. Aliquots of 50 µl of cell culture supernatant were reacted with equal volumes of Griess reagent (1% sulfanilamide, 0.1% naphthylenediamine dihydrochloride, 2.5% H_3_PO_4_) at room temperature (RT) for 10 min. Sodium nitrite was used to generate a standard curve for NO_2_^−^ production, and peak absorbance was measured at 550 nm with a Thermo_max_ microplate reader (Molecular Devices, Sunnyvale, CA). Cell-free medium contained <1.5 µM NO_2_^−^.

### Flow cytometry analysis of cell surface molecule activation by RAW264.7 cells

RAW264.7 cells were detached from 24-well culture plates, resuspended, and washed. Immunofluorescent staining for cell surface molecule expression was performed using the following fluorochrome-labeled mAbs from eBioscience (San Diego, CA), Biolegend (San Diego, CA), or BD Biosciences: CD11b (clone M1/70), CD80 (clone 16-10A1), CD40 (clone 3/23), TLR4 (clone MT5510), CD86 (clone GL1), TLR2 (clone T2.5) and MHC-II (clone AMS-32.1). Fluorescence was acquired on FACSCaliber, LSRII, or Canto (BD Biosciences). FlowJo (Tree Star, Ashland, OR) software was used for analysis.

### Isolation and infection of human macrophages co-cultured with autologous NK cells

Heparinized human peripheral blood was subjected to Histopaque 1077 (Sigma-Aldrich) density gradient centrifugation. The collected mononuclear cell fraction was collected, and monocytes were isolated with CD14 microbeads (Miltenyi Biotec, Auburn, CA) according to manufacturer's instructions. Monocytes (>95% purity,10^4^–10^5^/well) were then seeded into 48-well microtiter plates in CM without antibiotics, supplemented with 10 ng/ml GM-CSF (Peprotech, Rocky Hill, NJ) for 4–5 days at 37 C°/5% CO_2_ to generate macrophages. Human macrophages were infected with *F. tularensis* LVS (MOI∼300) or SchuS4 (MOI∼30) in the same manner as described above for murine macrophages. One day prior to macrophage infection, autologous “untouched” NK cells were isolated from human PBMCs using an NK cell isolation kit from Miltenyi Biotec according to manufactures instructions. Isolated NK cells (>95% purity) were cultured overnight in complete media at 37 C°/5%CO_2_ with or without agonist stimulation. NK cells were washed with fresh CM prior to being added to wells containing infected autologous macrophages (∼2–20 NK cell/macrophage).

To inhibit the effects of IFN–γ *in vitro*, a neutralizing mAb (IFN–γ [clone B27, 1 µg/ml] in a no azide/low endotoxin (NA/LE) format was purchased from BD Biosciences and added to selected wells containing *Francisella*-infected macrophages with or without NK cells [60].

### Extraction of RNA and RT-PCR analysis of human NK cells

Human NK cells cultured with or without LVS-infected macrophages and/or Acai PS were centrifuged and resuspended in RNAlater reagent (Qiagen, Valencia, CA) until RNA extraction. Cells were then centrifuged and resuspended in Qiagen RLT buffer prior to lysis on a Qiashredder Column (Qiagen) and RNA extraction with an RNeasy Mini Kit (Qiagen). cDNA was generated using the Superscript III First Strand Synthesis System (Invitrogen). Primers for immune-related genes (TNF-α, IFN-γ, IL-17A, IL-21, IL-22, granzyme B, perforin, and TRAIL), along with β-actin (endogenous control), were designed using the PrimerQuest application from IDTDNA.com. The reference sequences used to generate these primers are listed below (paragraph “Accession numbers”). Amplicons were visualized under UV illumination on a 2% agarose gel containing GelRed (Biotium, Hayward, CA).

### Mouse infection, agonist treatment, *in vivo* neutralization, and CFU determination

Mice were infected with *F. tularensis* SchuS4 at Colorado State University via a whole-body low-dose aerosol, as previously described [53], [61]. Conscious mice within a stainless steel basket were exposed to the SchuS4 strain of *F. tularensis* by aerosol exposure in a Glascol Inhalation Exposure System (Glas-Col, Inc., Terre Haute, IN, USA). Prior to exposure, the nebulizer was loaded with bacteria diluted in PBS to a concentration of ∼5×10^6^ CFU/ml. Mice were exposed to a total of ∼4×10^7^ bacteria, aerosolized into a volume of 5 cubic feet over a period of 30 min, followed by a 20 min period of cloud decay in which airflow was maintained without introducing additional bacteria. This inoculum method generally delivers ∼50 CFUs of *F. tularensis* to the lungs of exposed mice and routinely results in 100% mortality and a mean time to death of 5–6 days following infection [53]. Mice infected with *F. tularensis* SchuS4 at Montana State University were infected with a 20 µl nasal volume (50 CFUs) placed onto the anterior nares following anesthesia induced by intraperitoneal (i.p.) injection of 100 µl of ketamine (12.5 mg/ml)+xylazine (3.8 mg/ml). For survival experiments, mice were monitored for morbidity and mortality twice daily for up to 28 days, at which time survivors were euthanized. Mice were treated with varying doses of Acai PS (in PBS) before or after infection. Mice were treated nasally under anesthesia (10 µl/nare induced by i.p. injection with ketamine/xylazine cocktail. For oral treatments, mice received 200 µl volume via gavage. Control mice were inoculated with PBS. For *in vivo* neutralization studies, mice were treated with 500 µg of mAb i.p. to neutralize IFN-γ (clone XMG 1.2) on day −2, while control mice received rat IgG [62]. In some experiments, mice were sacrificed 2 days post post-infection for CFU determination in lungs and spleens. Mouse organs were homogenized in sterile PBS, and homogenates were serially diluted and plated on MMH plates, which were then incubated at 37°C for 48 h, at which time CFUs were enumerated.

For *B. pseudomallei* infection, mice under ketamine/xylazine-induced anesthesia were infected with i.n. (10 µl/nare) with 3×10^3^ or 1×10^4^ CFUs of *B. pseudomallei* 1026b. Clinical scores were graded as 0 = normal; 1 = slightly ruffled; 2 = ruffled, sick looking; 3 = hunched posture and obviously ill; 4 = moribund; 5 = euthanized. For *in vivo* neutralization studies, mice received 500 µg of mAb i.p. to neutralize IFN-γ (clone XMG 1.2) or NK cells (clone PK136) on day −2, while control mice received rat IgG [62], [63]. Mice were sacrificed at 3 days post-infection for CFU determination in lungs, livers and spleens. Mouse organs were homogenized in sterile PBS, and homogenates were serial diluted and plated on Tryptic Soy Agar (BD Biosciences) plates, which were then incubated at 37°C for 48 h, at which time CFUs were enumerated.

### Pulmonary leukocyte activation assay

For *Francisella* studies, C57BL/6 mice were nasally treated with Acai PS 24 h prior to i.n. infection with 50 CFUs of *F. tularensis* SchuS4. Forty-eight h after infection, lung tissue was minced followed by digestion for 1 h at 37°C in CM containing 200 U/ml collagenase, (Sigma) and 0.08 U/ml DNAse (Promega, Madison, WI). The resulting cell suspensions were filtered through 35 mm NitexH nylon mesh (Sefar America; Depew, NY) to remove tissue debris, washed in CM, resuspended in 30% Percoll (Pharmacia, Uppsala, Sweden) and layered onto 70% Percoll, and subjected to density gradient centrifugation. Mononuclear cells were removed from the interface layer, washed, resuspended in CM, and cultured for 4 h in the presence of 12-myristate 13-acetate (PMA; 50 ng/ml), 500 ng/ml ionomycin, and 10 µg/ml brefeldin A. Cells were then analyzed by FACS analysis using conventional methods [64], [65]. Cells were stained for extracellular markers with fluorochrome-conjugated mAbs (Becton Dickinson or eBioscience, San Diego, CA): anti-NK1.1 (clone PK136); prior to fixation with 2% paraformaldehyde. Cells were then permeabilized with 0.2% saponin and stained for intracellular expression of IFN-γ (clone XMG1.2). Stained leukocytes were analyzed using an LSRII flow cytometer (BD Biosciences) and analyzed using FlowJo software (Tree Star Inc., Ashland, OR).

For *Burkholderia* studies, C57BL/6 mice were i.n. treated with Acai PS 24 h prior to i.n. infection with 5×10^5^ CFUs of *B. thailandensis* E264. Twenty-four h after infection, lung tissue was processed, and cells were cultured and stained as described above.

### Statistical analysis

Statistical differences between two groups were determined using a Student's t test with the significance set at P<0.05. For comparison between three or more groups, analysis was done by one-way ANOVA followed by Tukey's multiple comparisons test with significance determined at P<0.05. For *in vivo* studies, significance in survival was assessed using log-rank analysis with significance set at P<0.05.

### Accession numbers

The GenBank (http://www.ncbi.nlm.nih.gov) accession numbers for DNA sequences utilized to generate primers are as follows: NM000594 (TNF-α), NM000619 (IFN-γ), NM002190 (IL-17A), NM021803 (IL-21), NM020525 (IL-22), NM004131 (granzyme B), FJ555237 (perforin), BC032722 (TRAIL), and NM001101 (β-actin).

## Supporting Information

Figure S1 **Acai PS confers time-, dose-, and NO-dependent protection against LVS infection of RAW264.7 cells.** A–C) RAW264.7 cells (10^6^/well, 3 wells/treatment) were infected with LVS. Some wells were stimulated with Acai PS before or after infection and/or treated with L-NMA (400 µM), an iNOS inhibitor. Twenty h after infection, cells were lysed and intracellular bacteria were enumerated A) and C). B) Nitrite levels in cell culture supernatants were measured; error bars represent SD. *P<0.05 as compared to untreated wells, ∧ P<0.05 as compared to overnight pretreatment with 100 µg Acai PS (-13 h) in A–B), and Acai PS given 8 h after infection in C). Results are representative of two independent experiments. NA = not applicable. D) RAW264.7 cells (10^6^/well, 3 wells/treatment) were infected with LVS. Some wells were stimulated with Acai PS (100 µg/ml) immediately after infection. At 4, 8, and 20 h after infection, cells were lysed, and intracellular bacteria were enumerated. E–G) NO and cytokine levels in cell culture supernatants were measured; error bars represent SD * P<0.05 as compared to untreated wells. Results are representative of two independent experiments.
(TIF)

Figure S2 **Prophylactic Acai PS immunotherapy is optimal for protection against high dose** ***B. pseudomallei*** **infection.** C57BL/6 mice (n = 10/group) were treated intranasally with 100 or 1000 µg of Acai PS one day prior to i.n. infection with 1×10^4^ CFUs of *B. pseudomallei* 1026b. A) Survival and B) clinical scores were monitored over time. Error bars depict SEM. *P<0.05 as compared to PBS group. **** indicates that *P<0.05 for all Acai PS-treated groups relative to the PBS group at the same time point.
(TIF)

Table S1 **Acai PS induces up-regulation of macrophage surface activation molecules in both mock- and LVS-infected RAW264.7 cells.** RAW264.7 macrophages (10^6^/well, 3 wells/treatment) were stimulated overnight (∼16 h) or not with Acai PS prior to infection with *F. tularensis* LVS (MOI∼300). After 20 h of infection, Median fluorescence intensity (MFI) mean from three wells/treatment was determined via flow cytometry. Standard error in parentheses; results are representative of two independent experiments. *P<0.05 as compared to cells not treated with Acai PS within same infection treatment.
(PDF)

Table S2 **Acai PS induces production of proinflammatory cytokines in both mock- and LVS-infected RAW264.7 cells.** RAW264.7 macrophages (10^6^/well, 3 wells/treatment) were stimulated overnight (∼16 h) or not with Acai PS prior to infection with *F. tularensis* LVS (MOI∼300). After 20 h of infection, the production of cytokines and NO was determined by ELISA or the Griess reaction. Standard error in parentheses; results are representative of two independent experiments. *P<0.05 as compared to cells not treated with Acai PS within same infection treatment.
(PDF)

Table S3 **Acai PS does not induce cytotoxicity in murine and human macrophages infected with type A** ***F. tularensis*** **.** RAW264.7 cells, murine BMDM, or human macrophages were treated or not with Acai PS 16 hr prior to infection with *F. tularensis* SchuS4 (MOI∼30). Cytotoxicity was measured by LDH release at 20 hr after infection and expressed as a percentage of LDH release by Triton X-100 detergent. Standard deviation in parentheses.
(PDF)

Table S4 **Oral administration of Acai PS confers variable protection against aerosol infection with** ***F. tularensis*** **SchuS4.** C57BL/6 mice were treated orally with PBS or Acai PS before or after aerosol infection with *F. tularensis* SchuS4, and survival was monitored over time.
(PDF)
