# Supplementary figures and images for: Nasal Acai Polysaccharides Potentiate Innate Immunity to Protect against Pulmonary Francisella tularensis and Burkholderia pseudomallei Infections
Source: PLoS Pathog. 2012 Mar 15;8(3):e1002587. doi: 10.1371/journal.ppat.1002587 (PMC3305411; doi:10.1371/journal.ppat.1002587)

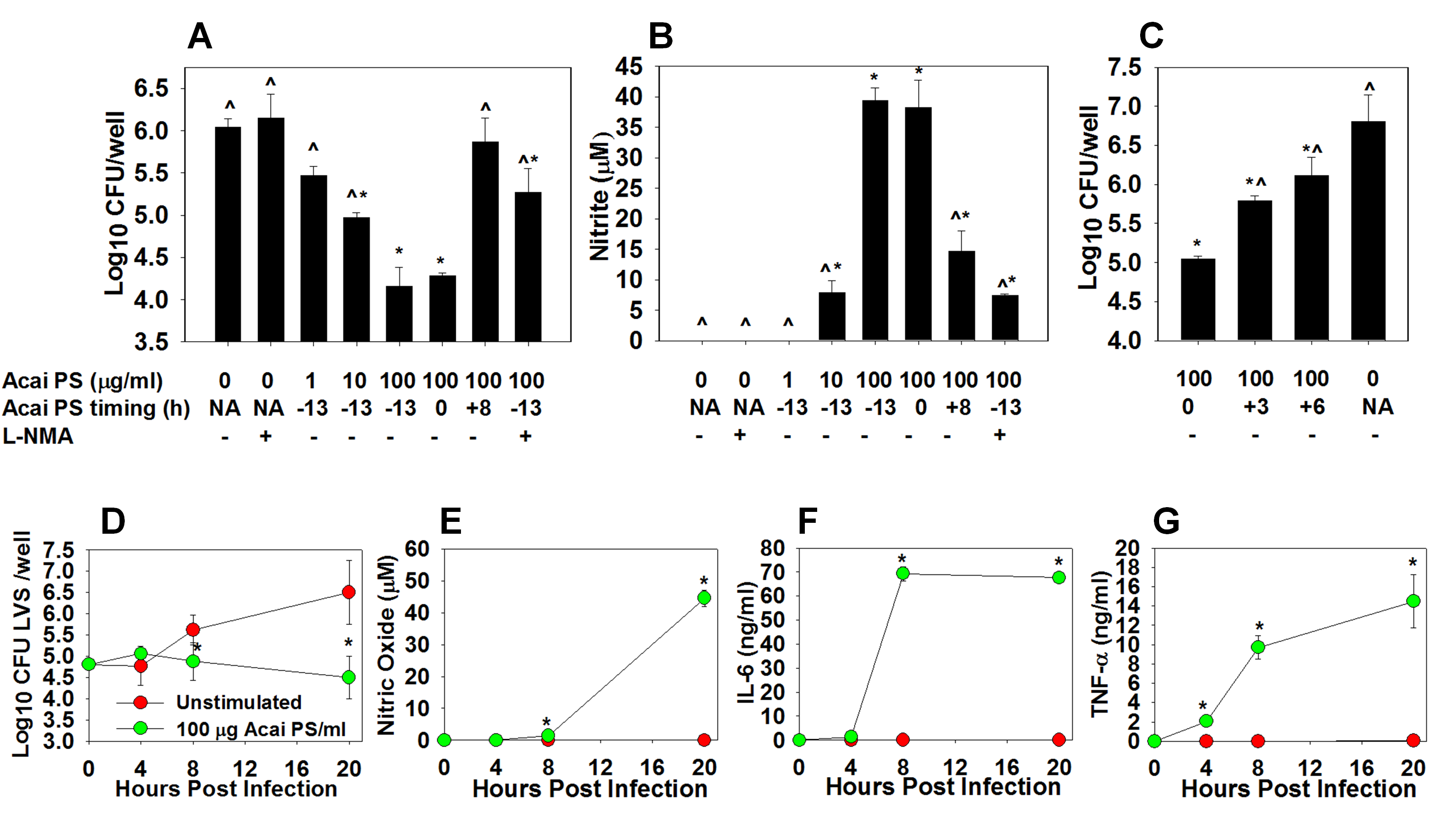

Supplement: Figure S1 — Acai PS confers time-, dose-, and NO-dependent protection against LVS infection of RAW264.7 cells. A–C) RAW264.7 cells (106/well, 3 wells/treatment) were infected with LVS. Some wells were stimulated with Acai PS before or after infection and/or treated with L-NMA (400 µM), an iNOS inhibitor. Twenty h after infection, cells were lysed and intracellular bacteria were enumerated A) and C). B) Nitrite levels in cell culture supernatants were measured; error bars represent SD. *P<0.05 as compared to untreated wells, ∧ P<0.05 as compared to overnight pretreatment with 100 µg Acai PS (-13 h) in A–B), and Acai PS given 8 h after infection in C). Results are representative of two independent experiments. NA = not applicable. D) RAW264.7 cells (106/well, 3 wells/treatment) were infected with LVS. Some wells were stimulated with Acai PS (100 µg/ml) immediately after infection. At 4, 8, and 20 h after infection, cells were lysed, and intracellular bacteria were enumerated. E–G) NO and cytokine levels in cell culture supernatants were measured; error bars represent SD * P<0.05 as compared to untreated wells. Results are representative of two independent experiments. (TIF) [file ppat.1002587.s001.tif]

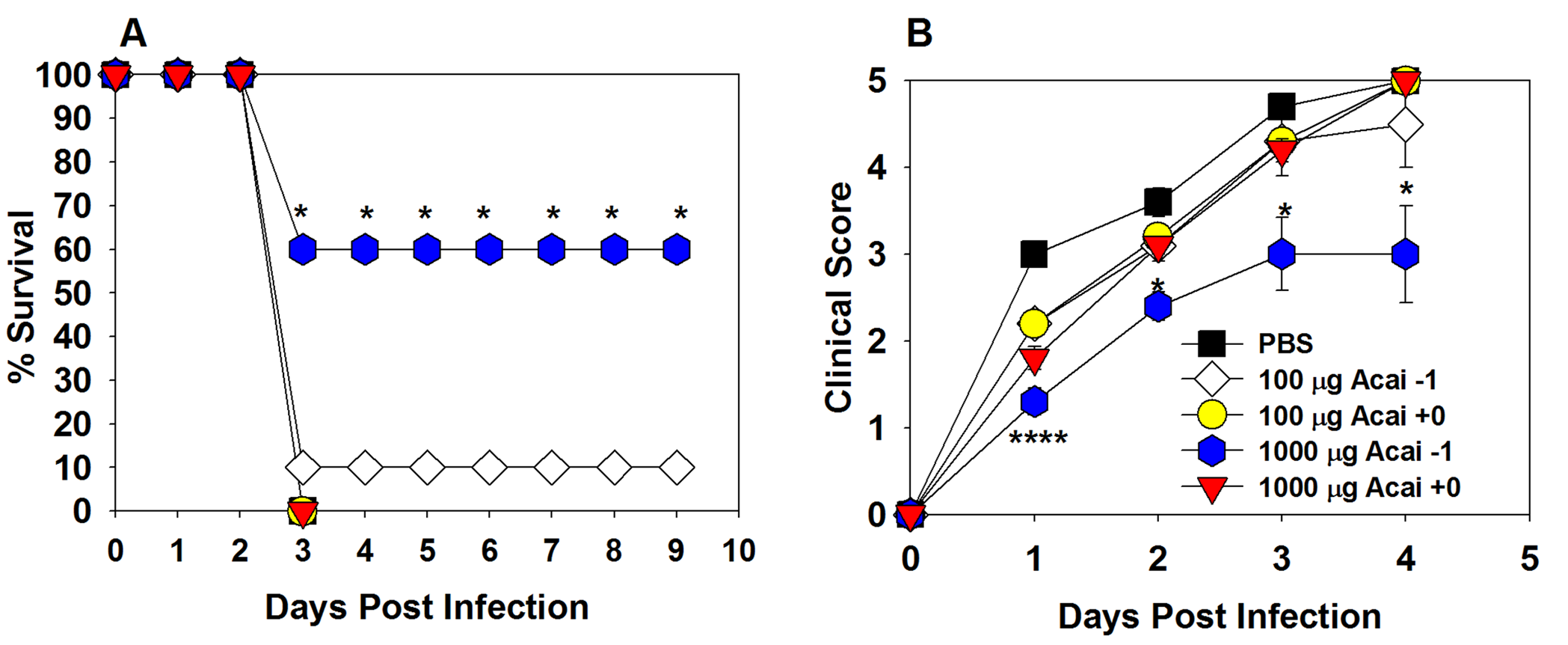

Supplement: Figure S2 — Prophylactic Acai PS immunotherapy is optimal for protection against high dose B. pseudomallei infection. C57BL/6 mice (n = 10/group) were treated intranasally with 100 or 1000 µg of Acai PS one day prior to i.n. infection with 1×10^4^ CFUs of B. pseudomallei 1026b. A) Survival and B) clinical scores were monitored over time. Error bars depict SEM. *P<0.05 as compared to PBS group. **** indicates that *P<0.05 for all Acai PS-treated groups relative to the PBS group at the same time point. (TIF) [file ppat.1002587.s002.tif]
