# Supplementary material for: Nasal Acai Polysaccharides Potentiate Innate Immunity to Protect against Pulmonary Francisella tularensis and Burkholderia pseudomallei Infections
Source: PLoS Pathog. 2012 Mar 15;8(3):e1002587. doi: 10.1371/journal.ppat.1002587 (PMC3305411; doi:10.1371/journal.ppat.1002587)
Supplement: Table S1 — Acai PS induces up-regulation of macrophage surface activation molecules in both mock- and LVS-infected RAW264.7 cells. RAW264.7 macrophages (106/well, 3 wells/treatment) were stimulated overnight (∼16 h) or not with Acai PS prior to infection with F. tularensis LVS (MOI∼300). After 20 h of infection, Median fluorescence intensity (MFI) mean from three wells/treatment was determined via flow cytometry. Standard error in parentheses; results are representative of two independent experiments. *P<0.05 as compared to cells not treated with Acai PS within same infection treatment. (PDF) [file ppat.1002587.s003.pdf]

Table S1. Acai PS induces up-regulation of macrophage surface activation molecules and in both mock- and LVS-infected RAW264.7 cells[a].

	Mock-infected cells[b] Acai PS concentration				LVS-infected cells[b] Acai PS concentration			
	**Media**	**1 µg/ml**	**10 µg/ml**	**100 µg/ml**	**Media**	**1 µg/ml**	**10 µg/ml**	**100 µg/ml**
CD11b	9417 (381)	9278 (42.0)	10767 (94.0)*	17334 (483)*	10195 (186)	10987 (467)	11280(989)	20108 (456)*
CD40	487 (11.6)	442 (31.6)	678 (19.2)*	2967 (303)*	682 (10.4)	699 (47.3)	750 (106)	3576 (126)*
CD80	477 (3.9)	473 (6.8)	556 (18.0)	1927 (239)*	726 (43.2)	692 (25.7)	877 (25.9)	3378 (181)*
CD86	1200 (22.4)	1170 (37.8)	1304 (95.0)*	3618 (213)*	1349 (60.0)	1316 (73.0)	1683 (118)*	4259 (75.2)*
TLR2	1156 (19.0)	1061 (16.0)*	1051 (32.0)	1205 (20.0)*	1308 (38.0)	1251 (31.0)	1281 (23.0)	1521 (11.8)*
TLR4	740 (29.9)	644 (29.3)	524 (16.8)*	578 (35.4)*	839 (48.3)	822 (41.1)	728 (44.5)	642 (49.0)*
MHCII	62.5 (3.25)	65.0 (3.13)	70.5 (2.59)	156.6 (12.8)	80.6 (3.6)	89.0 (2.96)	105 (4.75)	201 (7.70)

[a]Cells pretreated with Acai PS 16 hr prior to infection with LVS.
[b]Median fluorescence intensity (MFI) mean from three wells/treatment shown; standard error in parentheses; results are representative of two independent experiments.
*P<0.05 as compared to cells not treated with Acai PS within same infection treatment.
