# Supplementary material for: Nasal Acai Polysaccharides Potentiate Innate Immunity to Protect against Pulmonary Francisella tularensis and Burkholderia pseudomallei Infections
Source: PLoS Pathog. 2012 Mar 15;8(3):e1002587. doi: 10.1371/journal.ppat.1002587 (PMC3305411; doi:10.1371/journal.ppat.1002587)
Supplement: Table S2 — Acai PS induces production of proinflammatory cytokines in both mock- and LVS-infected RAW264.7 cells. RAW264.7 macrophages (106/well, 3 wells/treatment) were stimulated overnight (∼16 h) or not with Acai PS prior to infection with F. tularensis LVS (MOI∼300). After 20 h of infection, the production of cytokines and NO was determined by ELISA or the Griess reaction. Standard error in parentheses; results are representative of two independent experiments. *P<0.05 as compared to cells not treated with Acai PS within same infection treatment. (PDF) [file ppat.1002587.s004.pdf]

Table S2. Acai PS induces production of proinflammatory cytokines in both mock- and LVS-infected RAW264.7 cells[a].

	Mock-infected cells[b] Acai PS concentration				LVS-Infected cells[b] Acai PS concentration			
	**Media**	**1 µg/ml**	**10 µg/ml**	**100 µg/ml**	**Media**	**1 µg/ml**	**10 µg/ml**	**100 µg/ml**
NO[c]	0	0	0	17.1 (1.53)	0	0	6.4 (1.09)	35.8 (4.10)
TNF-α[d]	0	0	1.63 (0.285)*	8.65 (0.38)*	0	1.83 (0.43)*	2.06 (1.16)*	4.74 (0.9)*
IL-6[d]	0	0	0	89.2 (2.69)*	0.155 (0.04)	0.286 (.05)*	39.7 (5.26)*	91.17 (4.42)*

[b]Cells pretreated with Acai PS 16 hr prior to infection with LVS.
[c]Mean NO (µM) or [d]cytokine production from three wells/treatment shown; standard error in parentheses; results are representative of two independent experiments.
*P<0.05 as compared to cells not treated with Acai PS within same infection treatment.
