# Supplementary material for: Nasal Acai Polysaccharides Potentiate Innate Immunity to Protect against Pulmonary Francisella tularensis and Burkholderia pseudomallei Infections
Source: PLoS Pathog. 2012 Mar 15;8(3):e1002587. doi: 10.1371/journal.ppat.1002587 (PMC3305411; doi:10.1371/journal.ppat.1002587)
Supplement: Table S3 — Acai PS does not induce cytotoxicity in murine and human macrophages infected with type A F. tularensis . RAW264.7 cells, murine BMDM, or human macrophages were treated or not with Acai PS 16 hr prior to infection with F. tularensis SchuS4 (MOI∼30). Cytotoxicity was measured by LDH release at 20 hr after infection and expressed as a percentage of LDH release by Triton X-100 detergent. Standard deviation in parentheses. (PDF) [file ppat.1002587.s005.pdf]

Table S3. Acai PS does not induce cytotoxicity in murine and human macrophages infected with type A *F. tularensis*[a].

	% Cytotoxicity		
	**Media**	**10 µg/ml Acai PS**	**100 µg/ml Acai PS**
RAW264.7 Cells	7.57 (0.42)	8.72 (1.38)	7.68 (0.24)
Mouse BMDM	11.96 (0.71)	9.79 (0.12)	10.88 (2.83)
Human Macrophages	3.38 (0.14)	4.08 (1.62)	3.78 (0.44)

[a]Cells were treated or not with Acai PS 16 hr prior to infection with *F. tularensis* SchuS4 (MOI~30).
[b]Cytotoxicity was measured by LDH release at 20 hr after infection and expressed as a percentage of LDH release by Triton X-100 detergent.
Standard deviation in parentheses.
