# Supplementary material for: Nasal Acai Polysaccharides Potentiate Innate Immunity to Protect against Pulmonary Francisella tularensis and Burkholderia pseudomallei Infections
Source: PLoS Pathog. 2012 Mar 15;8(3):e1002587. doi: 10.1371/journal.ppat.1002587 (PMC3305411; doi:10.1371/journal.ppat.1002587)
Supplement: Table S4 — Oral administration of Acai PS confers variable protection against aerosol infection with F. tularensis SchuS4. C57BL/6 mice were treated orally with PBS or Acai PS before or after aerosol infection with F. tularensis SchuS4, and survival was monitored over time. (PDF) [file ppat.1002587.s006.pdf]

**Table S4.** Oral administration of Acai PS confers variable protection against aerosol infection with *F. tularensis* SchuS4[a].

| Treatment | Dose | Route | Timing (Day #) | Survival (Exp #1) | Survival (Exp #2) | Survival (Total) |
|---|---|---|---|---|---|---|
| PBS | | | | 0/10 (0%) | 0/10 (0%) | 0/20 (0%) |
| Acai PS | 100 µg | Oral | -1 | 1/5 (20%) | ND | 1/5 (20%) |
| Acai PS | 1 mg | Oral | -1 | 3/5 (60%) | 0/5 (0%) | 3/10 (20%) |
| Acai PS | 10 mg | Oral | -1 | 2/5 (40%) | ND | 2/5 (40%) |
| Acai PS | 1 mg | Oral | +0 | ND | 0/5 (0%) | 0/5 (0%) |
| Acai PS | 1 mg | Oral | +1 | ND | 1/5 (20%) | 1/5 (20%) |
| Acai PS | 1 mg | Oral | +2 | ND | 1/5 (20%) | 1/5 (20%) |

[a]Mice were treated orally with PBS or Acai PS before or after aerosol infection with *F. tularensis* SchuS4, and survival was monitored over time.
